# μ_3_-Bromido-oxidotri-μ_3_-sulfido-tris­(triphenyl­phosphane-κ*P*)­tri­copper(I)­tungsten(VI)

**DOI:** 10.1107/S1600536812004631

**Published:** 2012-02-10

**Authors:** Wenjiang Huang, Jinfang Zhang, Chi Zhang

**Affiliations:** a Institute of Molecular Engineering and Advanced Materials, School of Chemical Engineering, Nanjing University of Science and Technology, 200 Xiaolingwei, Nanjing 210094, Jiangsu, People’s Republic of China; b Institute of Science and Technology, Jiangsu University, 301 Xuefu Road, Zhenjiang 212013, People’s Republic of China

## Abstract

The title complex, [Cu_3_WBrOS_3_(C_18_H_15_P)_3_], a neutral heavily distorted cubane-like W/S/Cu cluster, was self-assembled from ammonium trithio­tungstate(VI), cuprous bromide and triphenyl­phosphane in *N*,*N*-dimethyl­formamide. The average Cu—Br, Cu—S and W—μ_3_-S bond lengths are 2.731 (2), 2.318 (2) and 2.256 (2) Å, respectively, in the distorted cubane-like skeleton. The W atom exhibits tetra­hedral geometry, formed by one terminal O atom and three μ_3_-S atoms; the W—O bond length is 1.728 (6) Å. Each Cu atom is coordinated by one P atom from a triphenyl­phosphane ligand, and two μ_3_-S and one μ_3_-Br atoms, forming a distorted tetra­hedral coordination geometry.

## Related literature

For heterothio­metallic Mo(W)/S/Cu(Ag) clusters and their unique properties, see: Zhang *et al.* (2007 ▶). For cubane-like Mo(W)/S/Cu(Ag) clusters, see: Tang *et al.* (2008 ▶); Müller *et al.* (1983 ▶).
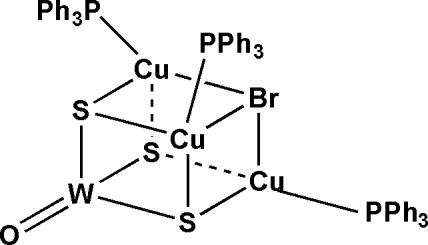


## Experimental

### 

#### Crystal data

[Cu_3_WBrOS_3_(C_18_H_15_P)_3_]
*M* *_r_* = 1353.41
Orthorhombic, 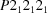

*a* = 12.8600 (13) Å
*b* = 17.6436 (17) Å
*c* = 22.732 (2) Å
*V* = 5157.8 (9) Å^3^
*Z* = 4
Mo *K*α radiation
μ = 4.47 mm^−1^
*T* = 293 K
0.35 × 0.18 × 0.15 mm

#### Data collection

Rigaku Saturn724+ (2 × 2 bin mode) diffractometer
Absorption correction: multi-scan (*CrystalClear*; Rigaku, 2007 ▶) *T*_min_ = 0.304, *T*_max_ = 0.554
49122 measured reflections
9409 independent reflections
8353 reflections with *I* > 2σ(*I*)
*R*_int_ = 0.075

#### Refinement

*R*[*F* ^2^ > 2σ(*F* ^2^)] = 0.047
*wR*(*F* ^2^) = 0.114
*S* = 1.10
9409 reflections
595 parameters
H-atom parameters constrained
Δρ_max_ = 0.78 e Å^−3^
Δρ_min_ = −0.97 e Å^−3^
Absolute structure: Flack (1983 ▶), 4199 Friedel pairs
Flack parameter: −0.014 (8)

### 

Data collection: *CrystalClear* (Rigaku, 2007 ▶); cell refinement: *CrystalClear*; data reduction: *CrystalClear*; program(s) used to solve structure: *SHELXS97* (Sheldrick, 2008 ▶); program(s) used to refine structure: *SHELXL97* (Sheldrick, 2008 ▶); molecular graphics: *SHELXTL* (Sheldrick, 2008 ▶); software used to prepare material for publication: *SHELXTL*.

## Supplementary Material

Crystal structure: contains datablock(s) I, global. DOI: 10.1107/S1600536812004631/pv2509sup1.cif

Structure factors: contains datablock(s) I. DOI: 10.1107/S1600536812004631/pv2509Isup2.hkl

Additional supplementary materials:  crystallographic information; 3D view; checkCIF report

## Figures and Tables

**Fig. 1.**
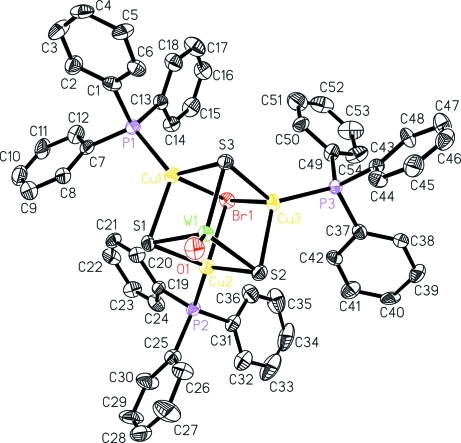
The molecular structure of the title compound, with atomic labels and 30% probability displacement ellipsoids. H atoms have been omitted for clarity.

## supplementary crystallographic information

### Comment

Heterothiometallic Mo(W)/S/Cu(Ag) clusters have attracted much attention for their configurational isomerism and unique properties as functional materials, such as third-order nonlinear optical (NLO) materials (Zhang *et al.*, 2007). Triphenylphosphane ligand has been proved effective to obtain the cubane-like Mo(W)/S/Cu(Ag) clusters (Tang *et al.*, 2008; Müller *et al.*, 1983). The title compound with a cubane-like skeleton was also prepared by triphenylphosphane ligand.

The title compound (Fig. 1) exhibits a heavily distorted cubane-like skeleton, in which the average Cu—Br, Cu—S and W-µ_3_-S distances are 2.731 (2), 2.318 (2) and 2.256 (2) Å, respectively. The W atom exhibits tetrahedral geometry, formed by three µ_3_-S and one terminal O atom; the W—O bond length is 1.728 (6) Å. Each Cu atom is coordinated by one P from a triphenylphosphane, two µ_3_-S and one µ_3_-Br atoms, forming a distorted tetrahedral coordination geometry. The title complex is isostructural with I (Tang *et al.*, 2008) and Cl (Müller *et al.*, 1983) isomorphs.

### Experimental

CuBr (3 mmol) and P(Ph)_3_ (3 mmol) were added to a solution of [NH_4_]_2_WOS_3_ (1 mmol in 8 ml dmf) with thorough stirring for 10 minutes. After filtration the orange filtrate was carefully laid on the surface with 12 ml *i*-PrOH. The orange block crystals were obtained after two weeks.

### Refinement

H atoms were positioned geometrically and refined with riding model, with C—H = 0.93 Å and *U*_iso_ = 1.2*U*_eq_. The Flack index to establish an absolute structure was -0.014 (8), using 4199 Friedel pairs of reflections in the refinement.

### Crystal data

**Table:**

[Cu_3_WBrOS_3_(C_18_H_15_P)_3_]	*F*(000) = 2664
*M**_r_* = 1353.41	*D*_x_ = 1.743 Mg m^−^^3^
Orthorhombic, *P*2_1_2_1_2_1_	Mo *K*α radiation, λ = 0.71070 Å
Hall symbol: P 2ac 2ab	Cell parameters from 20240 reflections
*a* = 12.8600 (13) Å	θ = 3.1–25.4°
*b* = 17.6436 (17) Å	µ = 4.47 mm^−^^1^
*c* = 22.732 (2) Å	*T* = 293 K
*V* = 5157.8 (9) Å^3^	Block, orange
*Z* = 4	0.35 × 0.18 × 0.15 mm

### Data collection

**Table:**

Rigaku Saturn724+ (2x2 bin mode) diffractometer	9409 independent reflections
Radiation source: fine-focus sealed tube	8353 reflections with *I* > 2σ(*I*)
Graphite monochromator	*R*_int_ = 0.075
ω scans	θ_max_ = 25.4°, θ_min_ = 3.1°
Absorption correction: multi-scan (*CrystalClear*; Rigaku, 2007)	*h* = −15→15
*T*_min_ = 0.304, *T*_max_ = 0.554	*k* = −21→20
49122 measured reflections	*l* = −26→27

### Refinement

**Table:**

Refinement on *F*^2^	Secondary atom site location: difference Fourier map
Least-squares matrix: full	Hydrogen site location: inferred from neighbouring sites
*R*[*F*^2^ > 2σ(*F*^2^)] = 0.047	H-atom parameters constrained
*wR*(*F*^2^) = 0.114	*w* = 1/[σ^2^(*F*_o_^2^) + (0.0498*P*)^2^] where *P* = (*F*_o_^2^ + 2*F*_c_^2^)/3
*S* = 1.10	(Δ/σ)_max_ = 0.002
9409 reflections	Δρ_max_ = 0.78 e Å^−^^3^
595 parameters	Δρ_min_ = −0.97 e Å^−^^3^
0 restraints	Absolute structure: Flack (1983), 4199 Friedel pairs
Primary atom site location: structure-invariant direct methods	Flack parameter: −0.014 (8)

### Special details

**Table:**

Geometry. All e.s.d.'s (except the e.s.d. in the dihedral angle between two l.s. planes) are estimated using the full covariance matrix. The cell e.s.d.'s are taken into account individually in the estimation of e.s.d.'s in distances, angles and torsion angles; correlations between e.s.d.'s in cell parameters are only used when they are defined by crystal symmetry. An approximate (isotropic) treatment of cell e.s.d.'s is used for estimating e.s.d.'s involving l.s. planes.
Refinement. Refinement of *F*^2^ against ALL reflections. The weighted *R*-factor *wR* and goodness of fit *S* are based on *F*^2^, conventional *R*-factors *R* are based on *F*, with *F* set to zero for negative *F*^2^. The threshold expression of *F*^2^ > σ(*F*^2^) is used only for calculating *R*-factors(gt) *etc*. and is not relevant to the choice of reflections for refinement. *R*-factors based on *F*^2^ are statistically about twice as large as those based on *F*, and *R*- factors based on ALL data will be even larger.

### Fractional atomic coordinates and isotropic or equivalent isotropic displacement parameters (Å^2^)

**Table:**

	*x*	*y*	*z*	*U*_iso_*/*U*_eq_	
W1	0.83490 (3)	0.506350 (18)	0.024773 (14)	0.03477 (11)	
Br1	0.53543 (7)	0.45034 (5)	0.00444 (4)	0.0430 (2)	
Cu1	0.70443 (10)	0.40250 (6)	0.07471 (5)	0.0456 (3)	
Cu2	0.70050 (10)	0.45598 (6)	−0.06093 (5)	0.0435 (3)	
Cu3	0.64891 (9)	0.57834 (6)	0.03452 (5)	0.0455 (3)	
S1	0.81731 (18)	0.38494 (11)	−0.00357 (10)	0.0385 (5)	
S2	0.7603 (2)	0.57884 (12)	−0.04490 (10)	0.0418 (6)	
S3	0.74272 (19)	0.52247 (12)	0.10902 (10)	0.0405 (5)	
P1	0.6429 (2)	0.30728 (13)	0.12923 (10)	0.0381 (6)	
P2	0.6532 (2)	0.40308 (12)	−0.14515 (9)	0.0377 (5)	
P3	0.52241 (19)	0.66390 (12)	0.03685 (10)	0.0366 (5)	
O1	0.9641 (5)	0.5296 (3)	0.0351 (3)	0.0555 (17)	
C1	0.7003 (8)	0.2967 (5)	0.2020 (4)	0.044 (2)	
C2	0.7111 (10)	0.2272 (6)	0.2280 (5)	0.062 (3)	
H2A	0.6905	0.1832	0.2087	0.074*	
C3	0.7539 (10)	0.2243 (7)	0.2841 (5)	0.073 (4)	
H3A	0.7621	0.1772	0.3018	0.087*	
C4	0.7848 (9)	0.2881 (7)	0.3149 (4)	0.063 (3)	
H4A	0.8134	0.2844	0.3524	0.075*	
C5	0.7714 (9)	0.3578 (6)	0.2877 (4)	0.056 (3)	
H5A	0.7892	0.4020	0.3076	0.068*	
C6	0.7324 (8)	0.3620 (6)	0.2319 (4)	0.050 (3)	
H6A	0.7270	0.4089	0.2135	0.060*	
C7	0.6599 (9)	0.2132 (5)	0.0970 (4)	0.043 (2)	
C8	0.7512 (9)	0.1951 (6)	0.0704 (5)	0.055 (3)	
H8A	0.8036	0.2313	0.0683	0.066*	
C9	0.7678 (11)	0.1255 (7)	0.0468 (6)	0.079 (4)	
H9A	0.8308	0.1143	0.0287	0.095*	
C10	0.6873 (13)	0.0692 (7)	0.0502 (5)	0.076 (4)	
H10A	0.6975	0.0211	0.0344	0.091*	
C11	0.5988 (12)	0.0869 (6)	0.0759 (6)	0.076 (4)	
H11A	0.5472	0.0501	0.0786	0.091*	
C12	0.5799 (9)	0.1569 (6)	0.0986 (5)	0.054 (3)	
H12A	0.5154	0.1681	0.1150	0.065*	
C13	0.5042 (7)	0.3164 (5)	0.1429 (4)	0.041 (2)	
C14	0.4350 (8)	0.3095 (5)	0.0960 (5)	0.047 (2)	
H14A	0.4601	0.2994	0.0585	0.057*	
C15	0.3311 (9)	0.3174 (6)	0.1048 (5)	0.062 (3)	
H15A	0.2858	0.3097	0.0735	0.075*	
C16	0.2926 (9)	0.3360 (6)	0.1576 (5)	0.060 (3)	
H16A	0.2213	0.3424	0.1625	0.072*	
C17	0.3595 (9)	0.3456 (7)	0.2052 (6)	0.071 (3)	
H17A	0.3330	0.3581	0.2421	0.086*	
C18	0.4652 (9)	0.3365 (6)	0.1975 (5)	0.057 (3)	
H18A	0.5103	0.3439	0.2290	0.069*	
C19	0.5934 (7)	0.3094 (5)	−0.1345 (4)	0.037 (2)	
C20	0.6000 (8)	0.2715 (5)	−0.0818 (4)	0.044 (2)	
H20A	0.6368	0.2928	−0.0507	0.053*	
C21	0.5527 (9)	0.2027 (5)	−0.0750 (5)	0.056 (3)	
H21A	0.5597	0.1774	−0.0393	0.067*	
C22	0.4966 (9)	0.1706 (6)	−0.1180 (5)	0.056 (3)	
H22A	0.4631	0.1246	−0.1117	0.067*	
C23	0.4891 (8)	0.2076 (6)	−0.1733 (5)	0.055 (3)	
H23A	0.4551	0.1845	−0.2047	0.067*	
C24	0.5319 (8)	0.2765 (5)	−0.1794 (4)	0.052 (3)	
H24A	0.5208	0.3034	−0.2140	0.062*	
C25	0.7627 (8)	0.3921 (5)	−0.1946 (4)	0.045 (2)	
C26	0.8444 (10)	0.4411 (7)	−0.1880 (5)	0.071 (3)	
H26A	0.8433	0.4779	−0.1587	0.085*	
C27	0.9307 (12)	0.4349 (10)	−0.2264 (8)	0.107 (5)	
H27A	0.9834	0.4707	−0.2234	0.128*	
C28	0.9404 (12)	0.3809 (9)	−0.2663 (6)	0.089 (5)	
H28A	0.9997	0.3772	−0.2896	0.107*	
C29	0.8594 (13)	0.3302 (8)	−0.2722 (5)	0.090 (5)	
H29A	0.8642	0.2912	−0.2996	0.108*	
C30	0.7722 (11)	0.3366 (7)	−0.2382 (5)	0.077 (4)	
H30A	0.7175	0.3031	−0.2442	0.092*	
C31	0.5548 (9)	0.4496 (5)	−0.1901 (4)	0.045 (2)	
C32	0.5681 (9)	0.4705 (6)	−0.2469 (5)	0.063 (3)	
H32A	0.6327	0.4646	−0.2647	0.076*	
C33	0.4849 (15)	0.5011 (9)	−0.2791 (7)	0.105 (6)	
H33A	0.4946	0.5159	−0.3180	0.126*	
C34	0.3884 (14)	0.5091 (8)	−0.2529 (8)	0.102 (5)	
H34A	0.3329	0.5289	−0.2742	0.122*	
C35	0.3753 (11)	0.4878 (8)	−0.1957 (7)	0.093 (5)	
H35A	0.3106	0.4924	−0.1778	0.112*	
C36	0.4580 (10)	0.4596 (7)	−0.1649 (5)	0.066 (3)	
H36A	0.4487	0.4466	−0.1256	0.079*	
C37	0.4905 (7)	0.6990 (5)	−0.0361 (4)	0.041 (2)	
C38	0.4660 (9)	0.7721 (5)	−0.0496 (4)	0.050 (3)	
H38A	0.4668	0.8077	−0.0194	0.060*	
C39	0.4403 (10)	0.7966 (6)	−0.1053 (5)	0.065 (3)	
H39A	0.4237	0.8470	−0.1126	0.079*	
C40	0.4401 (9)	0.7430 (6)	−0.1499 (4)	0.059 (3)	
H40A	0.4243	0.7574	−0.1882	0.071*	
C41	0.4628 (10)	0.6701 (7)	−0.1377 (5)	0.064 (3)	
H41A	0.4611	0.6345	−0.1679	0.077*	
C42	0.4883 (8)	0.6469 (6)	−0.0819 (4)	0.048 (3)	
H42A	0.5040	0.5963	−0.0747	0.058*	
C43	0.5500 (7)	0.7499 (5)	0.0787 (4)	0.036 (2)	
C44	0.6493 (9)	0.7753 (5)	0.0815 (4)	0.047 (2)	
H44A	0.7026	0.7467	0.0650	0.057*	
C45	0.6723 (11)	0.8438 (6)	0.1090 (5)	0.068 (3)	
H45A	0.7403	0.8616	0.1103	0.081*	
C46	0.5969 (11)	0.8829 (6)	0.1328 (5)	0.064 (3)	
H46A	0.6125	0.9293	0.1501	0.077*	
C47	0.4981 (9)	0.8588 (6)	0.1334 (6)	0.068 (3)	
H47A	0.4472	0.8879	0.1516	0.081*	
C48	0.4712 (9)	0.7907 (5)	0.1071 (4)	0.050 (3)	
H48A	0.4033	0.7727	0.1082	0.060*	
C49	0.4001 (7)	0.6267 (5)	0.0624 (5)	0.046 (2)	
C50	0.3988 (9)	0.5673 (6)	0.1020 (4)	0.053 (3)	
H50A	0.4610	0.5485	0.1169	0.064*	
C51	0.3068 (13)	0.5361 (7)	0.1193 (6)	0.083 (4)	
H51A	0.3082	0.4959	0.1458	0.100*	
C52	0.2139 (11)	0.5607 (8)	0.0997 (5)	0.072 (4)	
H52A	0.1520	0.5386	0.1121	0.087*	
C53	0.2150 (10)	0.6185 (9)	0.0616 (6)	0.086 (4)	
H53A	0.1519	0.6368	0.0476	0.104*	
C54	0.3056 (8)	0.6522 (7)	0.0422 (5)	0.067 (3)	
H54A	0.3027	0.6920	0.0155	0.080*	

### Atomic displacement parameters (Å^2^)

**Table:**

	*U*^11^	*U*^22^	*U*^33^	*U*^12^	*U*^13^	*U*^23^
W1	0.02796 (18)	0.03604 (17)	0.04030 (19)	−0.00171 (16)	−0.00288 (15)	−0.00148 (16)
Br1	0.0348 (5)	0.0472 (5)	0.0470 (5)	−0.0028 (4)	−0.0006 (4)	−0.0039 (4)
Cu1	0.0549 (8)	0.0381 (6)	0.0438 (6)	−0.0078 (6)	0.0062 (6)	0.0024 (5)
Cu2	0.0532 (8)	0.0417 (6)	0.0357 (6)	−0.0014 (6)	−0.0094 (5)	−0.0031 (5)
Cu3	0.0451 (7)	0.0458 (6)	0.0456 (6)	0.0155 (5)	0.0003 (6)	0.0013 (5)
S1	0.0369 (14)	0.0370 (10)	0.0416 (11)	0.0075 (10)	−0.0028 (10)	−0.0017 (9)
S2	0.0456 (15)	0.0372 (11)	0.0425 (13)	−0.0026 (10)	0.0016 (11)	0.0057 (10)
S3	0.0491 (15)	0.0399 (12)	0.0326 (11)	0.0011 (10)	−0.0049 (10)	−0.0032 (9)
P1	0.0376 (15)	0.0376 (12)	0.0392 (13)	−0.0043 (10)	0.0014 (11)	0.0032 (10)
P2	0.0445 (15)	0.0373 (11)	0.0312 (11)	−0.0007 (11)	−0.0040 (11)	0.0032 (9)
P3	0.0336 (13)	0.0378 (12)	0.0385 (13)	0.0081 (10)	0.0001 (10)	0.0033 (10)
O1	0.036 (4)	0.054 (4)	0.077 (5)	−0.009 (3)	−0.003 (4)	−0.003 (3)
C1	0.037 (6)	0.054 (6)	0.040 (5)	−0.012 (5)	0.007 (4)	−0.006 (4)
C2	0.084 (9)	0.047 (6)	0.054 (7)	0.000 (6)	−0.011 (6)	0.001 (5)
C3	0.097 (11)	0.066 (7)	0.055 (7)	−0.011 (7)	−0.019 (7)	0.025 (6)
C4	0.068 (8)	0.095 (9)	0.025 (5)	−0.003 (7)	−0.002 (5)	−0.001 (5)
C5	0.074 (8)	0.061 (7)	0.034 (5)	−0.015 (6)	−0.006 (5)	0.006 (5)
C6	0.042 (7)	0.058 (6)	0.049 (6)	−0.001 (5)	0.007 (5)	−0.006 (5)
C7	0.056 (7)	0.042 (5)	0.033 (5)	0.006 (5)	−0.006 (5)	0.004 (4)
C8	0.057 (7)	0.055 (6)	0.054 (6)	0.013 (5)	0.011 (6)	0.010 (5)
C9	0.083 (10)	0.080 (9)	0.075 (9)	0.029 (8)	0.017 (7)	0.012 (7)
C10	0.109 (12)	0.053 (7)	0.064 (7)	0.023 (8)	−0.015 (8)	0.002 (6)
C11	0.113 (12)	0.030 (6)	0.084 (9)	0.002 (6)	−0.001 (9)	0.008 (6)
C12	0.047 (7)	0.052 (6)	0.065 (7)	−0.006 (5)	−0.010 (6)	0.005 (5)
C13	0.037 (6)	0.041 (5)	0.046 (6)	0.000 (4)	0.003 (5)	0.000 (4)
C14	0.037 (6)	0.056 (6)	0.049 (6)	−0.008 (5)	0.000 (5)	0.004 (5)
C15	0.044 (7)	0.071 (7)	0.073 (8)	−0.005 (6)	−0.018 (7)	0.009 (6)
C16	0.037 (6)	0.070 (7)	0.073 (8)	−0.002 (5)	0.001 (6)	−0.003 (6)
C17	0.042 (7)	0.096 (9)	0.077 (8)	0.001 (6)	0.000 (6)	−0.024 (7)
C18	0.040 (6)	0.073 (7)	0.059 (7)	0.010 (5)	−0.007 (5)	−0.007 (6)
C19	0.044 (6)	0.032 (4)	0.033 (5)	−0.002 (4)	−0.003 (4)	0.001 (4)
C20	0.042 (6)	0.048 (5)	0.043 (5)	−0.008 (4)	−0.008 (5)	−0.005 (4)
C21	0.057 (7)	0.043 (5)	0.066 (7)	−0.006 (5)	0.018 (6)	0.021 (5)
C22	0.052 (7)	0.049 (6)	0.066 (7)	−0.025 (5)	0.016 (6)	−0.021 (6)
C23	0.045 (7)	0.065 (7)	0.056 (7)	−0.015 (5)	−0.005 (5)	0.001 (5)
C24	0.051 (7)	0.053 (6)	0.051 (6)	−0.006 (5)	−0.006 (5)	−0.027 (5)
C25	0.055 (7)	0.047 (5)	0.032 (5)	−0.005 (5)	−0.006 (5)	−0.005 (4)
C26	0.065 (8)	0.083 (8)	0.064 (7)	0.001 (7)	0.016 (7)	−0.005 (6)
C27	0.054 (9)	0.139 (14)	0.128 (14)	−0.007 (9)	0.037 (9)	−0.012 (12)
C28	0.079 (11)	0.114 (11)	0.074 (9)	0.023 (10)	0.030 (8)	0.040 (9)
C29	0.121 (14)	0.099 (10)	0.048 (7)	−0.007 (10)	0.040 (8)	−0.013 (7)
C30	0.101 (11)	0.070 (8)	0.060 (8)	−0.022 (7)	0.025 (8)	−0.028 (6)
C31	0.061 (7)	0.038 (5)	0.036 (5)	−0.009 (5)	−0.020 (5)	0.000 (4)
C32	0.065 (8)	0.061 (7)	0.064 (7)	−0.012 (6)	−0.025 (6)	0.008 (5)
C33	0.140 (15)	0.084 (9)	0.092 (10)	−0.016 (12)	−0.074 (11)	0.032 (9)
C34	0.121 (13)	0.067 (8)	0.117 (12)	0.013 (10)	−0.075 (11)	0.019 (9)
C35	0.078 (10)	0.097 (10)	0.105 (11)	0.032 (8)	−0.043 (8)	−0.015 (9)
C36	0.066 (8)	0.075 (7)	0.056 (7)	0.021 (7)	−0.013 (6)	−0.011 (6)
C37	0.039 (6)	0.043 (5)	0.041 (5)	0.006 (4)	−0.003 (4)	−0.005 (4)
C38	0.065 (7)	0.044 (5)	0.042 (5)	0.003 (5)	0.014 (5)	−0.001 (4)
C39	0.076 (9)	0.058 (6)	0.062 (7)	0.018 (6)	−0.004 (7)	0.025 (6)
C40	0.080 (9)	0.078 (8)	0.019 (5)	−0.004 (7)	−0.001 (5)	0.004 (5)
C41	0.073 (9)	0.072 (8)	0.048 (6)	−0.005 (7)	−0.013 (6)	0.011 (6)
C42	0.060 (7)	0.050 (6)	0.035 (5)	0.004 (5)	−0.001 (5)	−0.010 (4)
C43	0.029 (5)	0.044 (5)	0.035 (5)	0.005 (4)	0.000 (4)	0.003 (4)
C44	0.044 (7)	0.054 (6)	0.044 (5)	−0.006 (5)	0.001 (5)	−0.009 (4)
C45	0.066 (9)	0.059 (7)	0.077 (8)	−0.007 (7)	−0.001 (7)	0.004 (6)
C46	0.084 (10)	0.050 (6)	0.057 (7)	−0.008 (7)	−0.021 (7)	0.006 (5)
C47	0.047 (8)	0.066 (7)	0.090 (9)	0.020 (6)	−0.010 (7)	−0.017 (6)
C48	0.041 (6)	0.045 (5)	0.065 (7)	0.009 (5)	0.002 (5)	−0.017 (5)
C49	0.032 (6)	0.046 (5)	0.060 (6)	−0.001 (4)	−0.002 (5)	0.009 (5)
C50	0.049 (7)	0.056 (6)	0.054 (6)	0.005 (5)	0.014 (5)	0.018 (5)
C51	0.101 (12)	0.063 (7)	0.085 (9)	−0.019 (8)	0.034 (9)	−0.009 (6)
C52	0.063 (9)	0.092 (9)	0.063 (8)	−0.038 (8)	0.014 (7)	−0.017 (7)
C53	0.052 (8)	0.137 (12)	0.070 (9)	−0.014 (9)	−0.008 (7)	0.001 (9)
C54	0.036 (7)	0.099 (8)	0.066 (7)	−0.012 (6)	0.000 (5)	0.027 (6)

### Geometric parameters (Å, º)

**Table:**

W1—O1	1.728 (6)	C19—C24	1.416 (12)
W1—S1	2.248 (2)	C20—C21	1.366 (13)
W1—S2	2.251 (2)	C20—H20A	0.9300
W1—S3	2.270 (2)	C21—C22	1.342 (15)
W1—Cu3	2.7173 (12)	C21—H21A	0.9300
W1—Cu1	2.7315 (12)	C22—C23	1.419 (15)
W1—Cu2	2.7519 (11)	C22—H22A	0.9300
Br1—Cu2	2.5932 (15)	C23—C24	1.342 (14)
Br1—Cu3	2.7744 (15)	C23—H23A	0.9300
Br1—Cu1	2.8262 (16)	C24—H24A	0.9300
Cu1—P1	2.232 (3)	C25—C26	1.368 (15)
Cu1—S3	2.309 (2)	C25—C30	1.398 (14)
Cu1—S1	2.317 (3)	C26—C27	1.417 (18)
Cu1—Cu2	3.2251 (15)	C26—H26A	0.9300
Cu1—Cu3	3.3120 (15)	C27—C28	1.32 (2)
Cu2—P2	2.215 (2)	C27—H27A	0.9300
Cu2—S2	2.329 (2)	C28—C29	1.379 (19)
Cu2—S1	2.351 (2)	C28—H28A	0.9300
Cu2—Cu3	3.1321 (15)	C29—C30	1.367 (18)
Cu3—P3	2.220 (2)	C29—H29A	0.9300
Cu3—S3	2.301 (2)	C30—H30A	0.9300
Cu3—S2	2.304 (3)	C31—C32	1.355 (14)
P1—C13	1.819 (10)	C31—C36	1.382 (15)
P1—C1	1.822 (10)	C32—C33	1.404 (17)
P1—C7	1.827 (9)	C32—H32A	0.9300
P2—C25	1.812 (10)	C33—C34	1.38 (2)
P2—C31	1.822 (10)	C33—H33A	0.9300
P2—C19	1.839 (9)	C34—C35	1.36 (2)
P3—C49	1.801 (10)	C34—H34A	0.9300
P3—C37	1.816 (9)	C35—C36	1.367 (16)
P3—C43	1.825 (9)	C35—H35A	0.9300
C1—C2	1.368 (14)	C36—H36A	0.9300
C1—C6	1.398 (13)	C37—C38	1.364 (12)
C2—C3	1.390 (15)	C37—C42	1.389 (12)
C2—H2A	0.9300	C38—C39	1.376 (14)
C3—C4	1.384 (16)	C38—H38A	0.9300
C3—H3A	0.9300	C39—C40	1.387 (15)
C4—C5	1.386 (15)	C39—H39A	0.9300
C4—H4A	0.9300	C40—C41	1.349 (15)
C5—C6	1.366 (14)	C40—H40A	0.9300
C5—H5A	0.9300	C41—C42	1.372 (14)
C6—H6A	0.9300	C41—H41A	0.9300
C7—C8	1.359 (14)	C42—H42A	0.9300
C7—C12	1.431 (14)	C43—C44	1.355 (13)
C8—C9	1.357 (16)	C43—C48	1.400 (13)
C8—H8A	0.9300	C44—C45	1.392 (14)
C9—C10	1.437 (18)	C44—H44A	0.9300
C9—H9A	0.9300	C45—C46	1.306 (17)
C10—C11	1.316 (18)	C45—H45A	0.9300
C10—H10A	0.9300	C46—C47	1.341 (16)
C11—C12	1.361 (15)	C46—H46A	0.9300
C11—H11A	0.9300	C47—C48	1.386 (15)
C12—H12A	0.9300	C47—H47A	0.9300
C13—C18	1.385 (14)	C48—H48A	0.9300
C13—C14	1.394 (13)	C49—C54	1.375 (14)
C14—C15	1.358 (15)	C49—C50	1.381 (13)
C14—H14A	0.9300	C50—C51	1.362 (16)
C15—C16	1.338 (15)	C50—H50A	0.9300
C15—H15A	0.9300	C51—C52	1.347 (19)
C16—C17	1.394 (16)	C51—H51A	0.9300
C16—H16A	0.9300	C52—C53	1.337 (18)
C17—C18	1.380 (15)	C52—H52A	0.9300
C17—H17A	0.9300	C53—C54	1.380 (17)
C18—H18A	0.9300	C53—H53A	0.9300
C19—C20	1.374 (12)	C54—H54A	0.9300
			
O1—W1—S1	111.2 (2)	C8—C9—C10	119.4 (12)
O1—W1—S2	111.8 (2)	C8—C9—H9A	120.3
S1—W1—S2	107.27 (8)	C10—C9—H9A	120.3
O1—W1—S3	111.0 (2)	C11—C10—C9	118.8 (11)
S1—W1—S3	107.97 (8)	C11—C10—H10A	120.6
S2—W1—S3	107.44 (9)	C9—C10—H10A	120.6
O1—W1—Cu3	136.4 (2)	C10—C11—C12	122.6 (13)
S1—W1—Cu3	112.34 (7)	C10—C11—H11A	118.7
S2—W1—Cu3	54.29 (7)	C12—C11—H11A	118.7
S3—W1—Cu3	54.06 (6)	C11—C12—C7	119.4 (11)
O1—W1—Cu1	133.9 (2)	C11—C12—H12A	120.3
S1—W1—Cu1	54.42 (6)	C7—C12—H12A	120.3
S2—W1—Cu1	114.31 (7)	C18—C13—C14	118.5 (9)
S3—W1—Cu1	54.03 (6)	C18—C13—P1	122.1 (8)
Cu3—W1—Cu1	74.87 (4)	C14—C13—P1	119.2 (7)
O1—W1—Cu2	141.0 (2)	C15—C14—C13	120.4 (10)
S1—W1—Cu2	54.99 (6)	C15—C14—H14A	119.8
S2—W1—Cu2	54.37 (6)	C13—C14—H14A	119.8
S3—W1—Cu2	108.04 (7)	C16—C15—C14	121.4 (11)
Cu3—W1—Cu2	69.87 (4)	C16—C15—H15A	119.3
Cu1—W1—Cu2	72.05 (4)	C14—C15—H15A	119.3
Cu2—Br1—Cu3	71.31 (4)	C15—C16—C17	119.8 (11)
Cu2—Br1—Cu1	72.90 (4)	C15—C16—H16A	120.1
Cu3—Br1—Cu1	72.50 (4)	C17—C16—H16A	120.1
P1—Cu1—S3	125.31 (10)	C18—C17—C16	119.7 (11)
P1—Cu1—S1	123.20 (9)	C18—C17—H17A	120.1
S3—Cu1—S1	104.38 (9)	C16—C17—H17A	120.1
P1—Cu1—W1	162.39 (9)	C17—C18—C13	120.0 (10)
S3—Cu1—W1	52.73 (6)	C17—C18—H18A	120.0
S1—Cu1—W1	52.10 (6)	C13—C18—H18A	120.0
P1—Cu1—Br1	105.43 (8)	C20—C19—C24	117.6 (8)
S3—Cu1—Br1	94.65 (7)	C20—C19—P2	121.7 (7)
S1—Cu1—Br1	95.03 (7)	C24—C19—P2	120.5 (7)
W1—Cu1—Br1	92.13 (4)	C21—C20—C19	120.3 (9)
P1—Cu1—Cu2	138.19 (8)	C21—C20—H20A	119.9
S3—Cu1—Cu2	93.33 (7)	C19—C20—H20A	119.9
S1—Cu1—Cu2	46.74 (6)	C22—C21—C20	122.2 (10)
W1—Cu1—Cu2	54.27 (3)	C22—C21—H21A	118.9
Br1—Cu1—Cu2	50.22 (3)	C20—C21—H21A	118.9
P1—Cu1—Cu3	141.42 (9)	C21—C22—C23	119.2 (9)
S3—Cu1—Cu3	43.98 (6)	C21—C22—H22A	120.4
S1—Cu1—Cu3	92.78 (6)	C23—C22—H22A	120.4
W1—Cu1—Cu3	52.37 (3)	C24—C23—C22	118.7 (10)
Br1—Cu1—Cu3	53.03 (3)	C24—C23—H23A	120.6
Cu2—Cu1—Cu3	57.24 (3)	C22—C23—H23A	120.6
P2—Cu2—S2	128.18 (9)	C23—C24—C19	121.8 (10)
P2—Cu2—S1	115.49 (9)	C23—C24—H24A	119.1
S2—Cu2—S1	101.45 (9)	C19—C24—H24A	119.1
P2—Cu2—Br1	104.74 (8)	C26—C25—C30	116.9 (11)
S2—Cu2—Br1	102.48 (8)	C26—C25—P2	117.4 (8)
S1—Cu2—Br1	100.64 (7)	C30—C25—P2	125.6 (9)
P2—Cu2—W1	157.00 (9)	C25—C26—C27	119.0 (12)
S2—Cu2—W1	51.78 (6)	C25—C26—H26A	120.5
S1—Cu2—W1	51.55 (6)	C27—C26—H26A	120.5
Br1—Cu2—W1	96.94 (4)	C28—C27—C26	123.6 (15)
P2—Cu2—Cu3	146.21 (8)	C28—C27—H27A	118.2
S2—Cu2—Cu3	47.14 (7)	C26—C27—H27A	118.2
S1—Cu2—Cu3	96.81 (6)	C27—C28—C29	117.6 (14)
Br1—Cu2—Cu3	57.04 (4)	C27—C28—H28A	121.2
W1—Cu2—Cu3	54.54 (3)	C29—C28—H28A	121.2
P2—Cu2—Cu1	135.03 (7)	C30—C29—C28	120.7 (12)
S2—Cu2—Cu1	96.75 (7)	C30—C29—H29A	119.6
S1—Cu2—Cu1	45.87 (6)	C28—C29—H29A	119.6
Br1—Cu2—Cu1	56.88 (4)	C29—C30—C25	122.1 (12)
W1—Cu2—Cu1	53.68 (3)	C29—C30—H30A	119.0
Cu3—Cu2—Cu1	62.78 (3)	C25—C30—H30A	119.0
P3—Cu3—S3	131.15 (10)	C32—C31—C36	118.3 (10)
P3—Cu3—S2	118.12 (9)	C32—C31—P2	124.7 (9)
S3—Cu3—S2	104.62 (10)	C36—C31—P2	116.8 (8)
P3—Cu3—W1	164.77 (8)	C31—C32—C33	120.3 (13)
S3—Cu3—W1	53.01 (6)	C31—C32—H32A	119.8
S2—Cu3—W1	52.47 (6)	C33—C32—H32A	119.8
P3—Cu3—Br1	100.02 (8)	C34—C33—C32	119.9 (14)
S3—Cu3—Br1	96.22 (7)	C34—C33—H33A	120.0
S2—Cu3—Br1	97.87 (7)	C32—C33—H33A	120.0
W1—Cu3—Br1	93.58 (4)	C35—C34—C33	119.5 (13)
P3—Cu3—Cu2	129.83 (8)	C35—C34—H34A	120.2
S3—Cu3—Cu2	95.94 (7)	C33—C34—H34A	120.2
S2—Cu3—Cu2	47.80 (6)	C34—C35—C36	119.5 (15)
W1—Cu3—Cu2	55.58 (3)	C34—C35—H35A	120.3
Br1—Cu3—Cu2	51.65 (3)	C36—C35—H35A	120.3
P3—Cu3—Cu1	142.04 (8)	C35—C36—C31	122.4 (12)
S3—Cu3—Cu1	44.17 (6)	C35—C36—H36A	118.8
S2—Cu3—Cu1	94.92 (7)	C31—C36—H36A	118.8
W1—Cu3—Cu1	52.76 (3)	C38—C37—C42	116.9 (9)
Br1—Cu3—Cu1	54.47 (3)	C38—C37—P3	125.5 (7)
Cu2—Cu3—Cu1	59.98 (3)	C42—C37—P3	117.6 (7)
W1—S1—Cu1	73.48 (7)	C37—C38—C39	124.1 (9)
W1—S1—Cu2	73.46 (6)	C37—C38—H38A	118.0
Cu1—S1—Cu2	87.39 (9)	C39—C38—H38A	118.0
W1—S2—Cu3	73.23 (7)	C38—C39—C40	117.3 (9)
W1—S2—Cu2	73.85 (7)	C38—C39—H39A	121.3
Cu3—S2—Cu2	85.07 (9)	C40—C39—H39A	121.3
W1—S3—Cu3	72.94 (7)	C41—C40—C39	119.9 (9)
W1—S3—Cu1	73.24 (7)	C41—C40—H40A	120.0
Cu3—S3—Cu1	91.85 (9)	C39—C40—H40A	120.0
C13—P1—C1	104.5 (4)	C40—C41—C42	121.8 (11)
C13—P1—C7	105.5 (5)	C40—C41—H41A	119.1
C1—P1—C7	102.9 (4)	C42—C41—H41A	119.1
C13—P1—Cu1	112.1 (3)	C41—C42—C37	120.0 (10)
C1—P1—Cu1	116.0 (3)	C41—C42—H42A	120.0
C7—P1—Cu1	114.8 (3)	C37—C42—H42A	120.0
C25—P2—C31	103.9 (5)	C44—C43—C48	119.3 (9)
C25—P2—C19	108.1 (4)	C44—C43—P3	118.9 (7)
C31—P2—C19	100.9 (4)	C48—C43—P3	121.8 (7)
C25—P2—Cu2	111.6 (3)	C43—C44—C45	120.6 (11)
C31—P2—Cu2	119.0 (3)	C43—C44—H44A	119.7
C19—P2—Cu2	112.3 (3)	C45—C44—H44A	119.7
C49—P3—C37	102.8 (4)	C46—C45—C44	119.1 (13)
C49—P3—C43	107.7 (4)	C46—C45—H45A	120.5
C37—P3—C43	103.7 (4)	C44—C45—H45A	120.5
C49—P3—Cu3	113.6 (3)	C45—C46—C47	122.7 (12)
C37—P3—Cu3	112.0 (3)	C45—C46—H46A	118.7
C43—P3—Cu3	115.8 (3)	C47—C46—H46A	118.7
C2—C1—C6	119.9 (9)	C46—C47—C48	120.4 (11)
C2—C1—P1	121.6 (8)	C46—C47—H47A	119.8
C6—C1—P1	118.5 (8)	C48—C47—H47A	119.8
C1—C2—C3	117.9 (10)	C47—C48—C43	117.7 (10)
C1—C2—H2A	121.0	C47—C48—H48A	121.2
C3—C2—H2A	121.0	C43—C48—H48A	121.2
C4—C3—C2	123.3 (11)	C54—C49—C50	117.1 (10)
C4—C3—H3A	118.4	C54—C49—P3	123.0 (8)
C2—C3—H3A	118.4	C50—C49—P3	119.9 (8)
C3—C4—C5	117.3 (10)	C51—C50—C49	120.3 (12)
C3—C4—H4A	121.3	C51—C50—H50A	119.8
C5—C4—H4A	121.3	C49—C50—H50A	119.8
C6—C5—C4	120.5 (10)	C52—C51—C50	123.0 (13)
C6—C5—H5A	119.7	C52—C51—H51A	118.5
C4—C5—H5A	119.7	C50—C51—H51A	118.5
C5—C6—C1	121.0 (10)	C53—C52—C51	116.7 (12)
C5—C6—H6A	119.5	C53—C52—H52A	121.6
C1—C6—H6A	119.5	C51—C52—H52A	121.6
C8—C7—C12	118.0 (9)	C52—C53—C54	123.0 (13)
C8—C7—P1	119.7 (8)	C52—C53—H53A	118.5
C12—C7—P1	122.3 (8)	C54—C53—H53A	118.5
C9—C8—C7	121.7 (12)	C49—C54—C53	119.9 (11)
C9—C8—H8A	119.2	C49—C54—H54A	120.1
C7—C8—H8A	119.2	C53—C54—H54A	120.1
			
O1—W1—Cu1—P1	−5.1 (4)	Cu1—Cu2—S1—W1	−73.53 (6)
S1—W1—Cu1—P1	80.9 (3)	P2—Cu2—S1—Cu1	−129.01 (10)
S2—W1—Cu1—P1	175.3 (3)	S2—Cu2—S1—Cu1	88.31 (9)
S3—W1—Cu1—P1	−90.2 (3)	Br1—Cu2—S1—Cu1	−16.90 (7)
Cu3—W1—Cu1—P1	−146.5 (3)	W1—Cu2—S1—Cu1	73.53 (6)
Cu2—W1—Cu1—P1	140.2 (3)	Cu3—Cu2—S1—Cu1	40.75 (7)
O1—W1—Cu1—S3	85.1 (3)	O1—W1—S2—Cu3	−132.4 (2)
S1—W1—Cu1—S3	171.06 (11)	S1—W1—S2—Cu3	105.48 (8)
S2—W1—Cu1—S3	−94.51 (10)	S3—W1—S2—Cu3	−10.40 (9)
Cu3—W1—Cu1—S3	−56.28 (8)	Cu1—W1—S2—Cu3	47.35 (7)
Cu2—W1—Cu1—S3	−129.60 (8)	Cu2—W1—S2—Cu3	89.64 (7)
O1—W1—Cu1—S1	−85.9 (3)	O1—W1—S2—Cu2	138.0 (2)
S2—W1—Cu1—S1	94.44 (10)	S1—W1—S2—Cu2	15.84 (9)
S3—W1—Cu1—S1	−171.06 (11)	S3—W1—S2—Cu2	−100.04 (8)
Cu3—W1—Cu1—S1	132.66 (8)	Cu3—W1—S2—Cu2	−89.64 (7)
Cu2—W1—Cu1—S1	59.35 (8)	Cu1—W1—S2—Cu2	−42.29 (8)
O1—W1—Cu1—Br1	179.4 (3)	P3—Cu3—S2—W1	165.69 (9)
S1—W1—Cu1—Br1	−94.71 (8)	S3—Cu3—S2—W1	10.11 (9)
S2—W1—Cu1—Br1	−0.28 (8)	Br1—Cu3—S2—W1	−88.47 (6)
S3—W1—Cu1—Br1	94.23 (8)	Cu2—Cu3—S2—W1	−74.60 (6)
Cu3—W1—Cu1—Br1	37.95 (4)	Cu1—Cu3—S2—W1	−33.70 (6)
Cu2—W1—Cu1—Br1	−35.37 (4)	P3—Cu3—S2—Cu2	−119.71 (10)
O1—W1—Cu1—Cu2	−145.3 (3)	S3—Cu3—S2—Cu2	84.71 (9)
S1—W1—Cu1—Cu2	−59.35 (8)	W1—Cu3—S2—Cu2	74.60 (6)
S2—W1—Cu1—Cu2	35.09 (7)	Br1—Cu3—S2—Cu2	−13.87 (7)
S3—W1—Cu1—Cu2	129.60 (8)	Cu1—Cu3—S2—Cu2	40.90 (7)
Cu3—W1—Cu1—Cu2	73.31 (4)	P2—Cu2—S2—W1	−150.60 (12)
O1—W1—Cu1—Cu3	141.4 (3)	S1—Cu2—S2—W1	−14.73 (9)
S1—W1—Cu1—Cu3	−132.66 (8)	Br1—Cu2—S2—W1	89.03 (6)
S2—W1—Cu1—Cu3	−38.22 (7)	Cu3—Cu2—S2—W1	73.95 (7)
S3—W1—Cu1—Cu3	56.28 (8)	Cu1—Cu2—S2—W1	31.53 (6)
Cu2—W1—Cu1—Cu3	−73.31 (4)	P2—Cu2—S2—Cu3	135.44 (12)
Cu2—Br1—Cu1—P1	−140.92 (8)	S1—Cu2—S2—Cu3	−88.68 (9)
Cu3—Br1—Cu1—P1	143.83 (8)	Br1—Cu2—S2—Cu3	15.08 (8)
Cu2—Br1—Cu1—S3	90.47 (7)	W1—Cu2—S2—Cu3	−73.95 (7)
Cu3—Br1—Cu1—S3	15.22 (7)	Cu1—Cu2—S2—Cu3	−42.42 (7)
Cu2—Br1—Cu1—S1	−14.44 (6)	O1—W1—S3—Cu3	132.9 (2)
Cu3—Br1—Cu1—S1	−89.69 (7)	S1—W1—S3—Cu3	−104.98 (8)
Cu2—Br1—Cu1—W1	37.69 (4)	S2—W1—S3—Cu3	10.43 (9)
Cu3—Br1—Cu1—W1	−37.56 (4)	Cu1—W1—S3—Cu3	−97.34 (8)
Cu3—Br1—Cu1—Cu2	−75.25 (4)	Cu2—W1—S3—Cu3	−46.90 (7)
Cu2—Br1—Cu1—Cu3	75.25 (4)	O1—W1—S3—Cu1	−129.8 (2)
Cu3—Br1—Cu2—P2	−148.60 (8)	S1—W1—S3—Cu1	−7.64 (9)
Cu1—Br1—Cu2—P2	134.57 (7)	S2—W1—S3—Cu1	107.77 (8)
Cu3—Br1—Cu2—S2	−13.14 (7)	Cu3—W1—S3—Cu1	97.34 (8)
Cu1—Br1—Cu2—S2	−89.96 (7)	Cu2—W1—S3—Cu1	50.44 (7)
Cu3—Br1—Cu2—S1	91.25 (7)	P3—Cu3—S3—W1	−161.08 (11)
Cu1—Br1—Cu2—S1	14.43 (6)	S2—Cu3—S3—W1	−10.04 (9)
Cu3—Br1—Cu2—W1	39.17 (4)	Br1—Cu3—S3—W1	89.79 (6)
Cu1—Br1—Cu2—W1	−37.66 (4)	Cu2—Cu3—S3—W1	37.83 (6)
Cu1—Br1—Cu2—Cu3	−76.82 (4)	Cu1—Cu3—S3—W1	71.83 (7)
Cu3—Br1—Cu2—Cu1	76.82 (4)	P3—Cu3—S3—Cu1	127.09 (12)
O1—W1—Cu2—P2	18.3 (4)	S2—Cu3—S3—Cu1	−81.87 (10)
S1—W1—Cu2—P2	−62.3 (2)	W1—Cu3—S3—Cu1	−71.83 (7)
S2—W1—Cu2—P2	99.1 (2)	Br1—Cu3—S3—Cu1	17.96 (8)
S3—W1—Cu2—P2	−162.01 (19)	Cu2—Cu3—S3—Cu1	−34.00 (8)
Cu3—W1—Cu2—P2	158.97 (19)	P1—Cu1—S3—W1	158.24 (11)
Cu1—W1—Cu2—P2	−121.02 (19)	S1—Cu1—S3—W1	7.28 (8)
O1—W1—Cu2—S2	−80.8 (3)	Br1—Cu1—S3—W1	−89.13 (6)
S1—W1—Cu2—S2	−161.45 (11)	Cu2—Cu1—S3—W1	−38.80 (6)
S3—W1—Cu2—S2	98.88 (10)	Cu3—Cu1—S3—W1	−71.56 (7)
Cu3—W1—Cu2—S2	59.86 (8)	P1—Cu1—S3—Cu3	−130.21 (12)
Cu1—W1—Cu2—S2	139.87 (9)	S1—Cu1—S3—Cu3	78.83 (10)
O1—W1—Cu2—S1	80.6 (3)	W1—Cu1—S3—Cu3	71.56 (7)
S2—W1—Cu2—S1	161.45 (11)	Br1—Cu1—S3—Cu3	−17.57 (8)
S3—W1—Cu2—S1	−99.67 (10)	Cu2—Cu1—S3—Cu3	32.76 (8)
Cu3—W1—Cu2—S1	−138.69 (8)	S3—Cu1—P1—C13	78.8 (4)
Cu1—W1—Cu2—S1	−58.68 (8)	S1—Cu1—P1—C13	−135.4 (3)
O1—W1—Cu2—Br1	178.8 (3)	W1—Cu1—P1—C13	156.0 (4)
S1—W1—Cu2—Br1	98.11 (8)	Br1—Cu1—P1—C13	−28.6 (3)
S2—W1—Cu2—Br1	−100.45 (9)	Cu2—Cu1—P1—C13	−75.2 (4)
S3—W1—Cu2—Br1	−1.56 (7)	Cu3—Cu1—P1—C13	20.5 (4)
Cu3—W1—Cu2—Br1	−40.58 (4)	S3—Cu1—P1—C1	−41.1 (4)
Cu1—W1—Cu2—Br1	39.42 (4)	S1—Cu1—P1—C1	104.7 (4)
O1—W1—Cu2—Cu3	−140.7 (3)	W1—Cu1—P1—C1	36.1 (5)
S1—W1—Cu2—Cu3	138.69 (8)	Br1—Cu1—P1—C1	−148.5 (4)
S2—W1—Cu2—Cu3	−59.86 (8)	Cu2—Cu1—P1—C1	164.9 (4)
S3—W1—Cu2—Cu3	39.02 (6)	Cu3—Cu1—P1—C1	−99.4 (4)
Cu1—W1—Cu2—Cu3	80.01 (4)	S3—Cu1—P1—C7	−161.0 (4)
O1—W1—Cu2—Cu1	139.3 (3)	S1—Cu1—P1—C7	−15.2 (4)
S1—W1—Cu2—Cu1	58.68 (8)	W1—Cu1—P1—C7	−83.8 (5)
S2—W1—Cu2—Cu1	−139.87 (9)	Br1—Cu1—P1—C7	91.7 (4)
S3—W1—Cu2—Cu1	−40.99 (6)	Cu2—Cu1—P1—C7	45.0 (4)
Cu3—W1—Cu2—Cu1	−80.01 (4)	Cu3—Cu1—P1—C7	140.8 (4)
P1—Cu1—Cu2—P2	−11.40 (19)	S2—Cu2—P2—C25	66.9 (4)
S3—Cu1—Cu2—P2	−170.38 (13)	S1—Cu2—P2—C25	−64.0 (4)
S1—Cu1—Cu2—P2	82.95 (14)	Br1—Cu2—P2—C25	−173.7 (3)
W1—Cu1—Cu2—P2	151.72 (13)	W1—Cu2—P2—C25	−13.8 (4)
Br1—Cu1—Cu2—P2	−77.10 (12)	Cu3—Cu2—P2—C25	134.5 (3)
Cu3—Cu1—Cu2—P2	−143.84 (13)	Cu1—Cu2—P2—C25	−116.1 (3)
P1—Cu1—Cu2—S2	166.23 (14)	S2—Cu2—P2—C31	−54.1 (4)
S3—Cu1—Cu2—S2	7.24 (10)	S1—Cu2—P2—C31	175.0 (4)
S1—Cu1—Cu2—S2	−99.43 (11)	Br1—Cu2—P2—C31	65.3 (4)
W1—Cu1—Cu2—S2	−30.66 (7)	W1—Cu2—P2—C31	−134.8 (4)
Br1—Cu1—Cu2—S2	100.52 (8)	Cu3—Cu2—P2—C31	13.5 (4)
Cu3—Cu1—Cu2—S2	33.78 (7)	Cu1—Cu2—P2—C31	122.9 (4)
P1—Cu1—Cu2—S1	−94.34 (15)	S2—Cu2—P2—C19	−171.6 (3)
S3—Cu1—Cu2—S1	106.67 (11)	S1—Cu2—P2—C19	57.5 (3)
W1—Cu1—Cu2—S1	68.77 (8)	Br1—Cu2—P2—C19	−52.2 (3)
Br1—Cu1—Cu2—S1	−160.05 (9)	W1—Cu2—P2—C19	107.7 (4)
Cu3—Cu1—Cu2—S1	133.21 (9)	Cu3—Cu2—P2—C19	−104.0 (3)
P1—Cu1—Cu2—Br1	65.71 (13)	Cu1—Cu2—P2—C19	5.4 (4)
S3—Cu1—Cu2—Br1	−93.28 (8)	S3—Cu3—P3—C49	−73.2 (4)
S1—Cu1—Cu2—Br1	160.05 (9)	S2—Cu3—P3—C49	138.9 (4)
W1—Cu1—Cu2—Br1	−131.18 (5)	W1—Cu3—P3—C49	−172.9 (4)
Cu3—Cu1—Cu2—Br1	−66.74 (4)	Br1—Cu3—P3—C49	34.3 (4)
P1—Cu1—Cu2—W1	−163.11 (13)	Cu2—Cu3—P3—C49	82.0 (4)
S3—Cu1—Cu2—W1	37.90 (7)	Cu1—Cu3—P3—C49	−8.6 (4)
S1—Cu1—Cu2—W1	−68.77 (8)	S3—Cu3—P3—C37	170.8 (3)
Br1—Cu1—Cu2—W1	131.18 (5)	S2—Cu3—P3—C37	22.9 (3)
Cu3—Cu1—Cu2—W1	64.44 (3)	W1—Cu3—P3—C37	71.2 (5)
P1—Cu1—Cu2—Cu3	132.45 (13)	Br1—Cu3—P3—C37	−81.7 (3)
S3—Cu1—Cu2—Cu3	−26.54 (7)	Cu2—Cu3—P3—C37	−34.0 (3)
S1—Cu1—Cu2—Cu3	−133.21 (9)	Cu1—Cu3—P3—C37	−124.6 (3)
W1—Cu1—Cu2—Cu3	−64.44 (3)	S3—Cu3—P3—C43	52.2 (3)
Br1—Cu1—Cu2—Cu3	66.74 (4)	S2—Cu3—P3—C43	−95.7 (3)
O1—W1—Cu3—P3	28.6 (5)	W1—Cu3—P3—C43	−47.5 (5)
S1—W1—Cu3—P3	−151.8 (3)	Br1—Cu3—P3—C43	159.7 (3)
S2—W1—Cu3—P3	−56.1 (3)	Cu2—Cu3—P3—C43	−152.6 (3)
S3—W1—Cu3—P3	111.7 (3)	Cu1—Cu3—P3—C43	116.8 (3)
Cu1—W1—Cu3—P3	167.9 (3)	C13—P1—C1—C2	88.2 (10)
Cu2—W1—Cu3—P3	−116.0 (3)	C7—P1—C1—C2	−21.8 (10)
O1—W1—Cu3—S3	−83.1 (4)	Cu1—P1—C1—C2	−147.9 (8)
S1—W1—Cu3—S3	96.55 (9)	C13—P1—C1—C6	−90.9 (8)
S2—W1—Cu3—S3	−167.72 (11)	C7—P1—C1—C6	159.1 (8)
Cu1—W1—Cu3—S3	56.26 (7)	Cu1—P1—C1—C6	33.0 (9)
Cu2—W1—Cu3—S3	132.32 (8)	C6—C1—C2—C3	0.3 (17)
O1—W1—Cu3—S2	84.7 (4)	P1—C1—C2—C3	−178.8 (9)
S1—W1—Cu3—S2	−95.73 (10)	C1—C2—C3—C4	1 (2)
S3—W1—Cu3—S2	167.72 (11)	C2—C3—C4—C5	0.2 (19)
Cu1—W1—Cu3—S2	−136.02 (8)	C3—C4—C5—C6	−2.1 (18)
Cu2—W1—Cu3—S2	−59.96 (8)	C4—C5—C6—C1	3.1 (17)
O1—W1—Cu3—Br1	−178.2 (3)	C2—C1—C6—C5	−2.2 (16)
S1—W1—Cu3—Br1	1.45 (7)	P1—C1—C6—C5	176.9 (9)
S2—W1—Cu3—Br1	97.18 (8)	C13—P1—C7—C8	166.1 (8)
S3—W1—Cu3—Br1	−95.10 (8)	C1—P1—C7—C8	−84.6 (8)
Cu1—W1—Cu3—Br1	−38.84 (4)	Cu1—P1—C7—C8	42.3 (9)
Cu2—W1—Cu3—Br1	37.22 (4)	C13—P1—C7—C12	−13.8 (9)
O1—W1—Cu3—Cu2	144.6 (3)	C1—P1—C7—C12	95.5 (8)
S1—W1—Cu3—Cu2	−35.77 (7)	Cu1—P1—C7—C12	−137.7 (7)
S2—W1—Cu3—Cu2	59.96 (8)	C12—C7—C8—C9	−1.3 (15)
S3—W1—Cu3—Cu2	−132.32 (8)	P1—C7—C8—C9	178.8 (9)
Cu1—W1—Cu3—Cu2	−76.06 (4)	C7—C8—C9—C10	−0.2 (18)
O1—W1—Cu3—Cu1	−139.3 (3)	C8—C9—C10—C11	0.3 (19)
S1—W1—Cu3—Cu1	40.29 (7)	C9—C10—C11—C12	1.1 (19)
S2—W1—Cu3—Cu1	136.02 (8)	C10—C11—C12—C7	−2.6 (18)
S3—W1—Cu3—Cu1	−56.26 (7)	C8—C7—C12—C11	2.6 (15)
Cu2—W1—Cu3—Cu1	76.06 (4)	P1—C7—C12—C11	−177.4 (9)
Cu2—Br1—Cu3—P3	133.59 (8)	C1—P1—C13—C18	19.1 (9)
Cu1—Br1—Cu3—P3	−149.05 (8)	C7—P1—C13—C18	127.2 (8)
Cu2—Br1—Cu3—S3	−92.67 (8)	Cu1—P1—C13—C18	−107.3 (8)
Cu1—Br1—Cu3—S3	−15.31 (7)	C1—P1—C13—C14	−166.6 (7)
Cu2—Br1—Cu3—S2	13.08 (7)	C7—P1—C13—C14	−58.5 (8)
Cu1—Br1—Cu3—S2	90.44 (7)	Cu1—P1—C13—C14	67.0 (8)
Cu2—Br1—Cu3—W1	−39.51 (4)	C18—C13—C14—C15	−4.3 (14)
Cu1—Br1—Cu3—W1	37.85 (4)	P1—C13—C14—C15	−178.8 (8)
Cu1—Br1—Cu3—Cu2	77.36 (4)	C13—C14—C15—C16	3.6 (16)
Cu2—Br1—Cu3—Cu1	−77.36 (4)	C14—C15—C16—C17	−1.7 (17)
P2—Cu2—Cu3—P3	−3.30 (19)	C15—C16—C17—C18	0.6 (18)
S2—Cu2—Cu3—P3	94.15 (13)	C16—C17—C18—C13	−1.3 (18)
S1—Cu2—Cu3—P3	−166.53 (11)	C14—C13—C18—C17	3.1 (16)
Br1—Cu2—Cu3—P3	−68.23 (10)	P1—C13—C18—C17	177.5 (9)
W1—Cu2—Cu3—P3	162.10 (11)	C25—P2—C19—C20	110.1 (8)
Cu1—Cu2—Cu3—P3	−134.73 (11)	C31—P2—C19—C20	−141.3 (8)
P2—Cu2—Cu3—S3	158.18 (15)	Cu2—P2—C19—C20	−13.4 (9)
S2—Cu2—Cu3—S3	−104.37 (11)	C25—P2—C19—C24	−74.9 (9)
S1—Cu2—Cu3—S3	−5.05 (10)	C31—P2—C19—C24	33.8 (9)
Br1—Cu2—Cu3—S3	93.25 (8)	Cu2—P2—C19—C24	161.6 (7)
W1—Cu2—Cu3—S3	−36.43 (7)	C24—C19—C20—C21	3.3 (15)
Cu1—Cu2—Cu3—S3	26.74 (7)	P2—C19—C20—C21	178.5 (8)
P2—Cu2—Cu3—S2	−97.45 (16)	C19—C20—C21—C22	−1.7 (17)
S1—Cu2—Cu3—S2	99.32 (11)	C20—C21—C22—C23	2.4 (17)
Br1—Cu2—Cu3—S2	−162.38 (9)	C21—C22—C23—C24	−4.9 (16)
W1—Cu2—Cu3—S2	67.95 (8)	C22—C23—C24—C19	6.9 (16)
Cu1—Cu2—Cu3—S2	131.12 (9)	C20—C19—C24—C23	−6.1 (15)
P2—Cu2—Cu3—W1	−165.40 (15)	P2—C19—C24—C23	178.7 (8)
S2—Cu2—Cu3—W1	−67.95 (8)	C31—P2—C25—C26	103.4 (9)
S1—Cu2—Cu3—W1	31.38 (6)	C19—P2—C25—C26	−150.0 (8)
Br1—Cu2—Cu3—W1	129.67 (4)	Cu2—P2—C25—C26	−26.1 (9)
Cu1—Cu2—Cu3—W1	63.17 (3)	C31—P2—C25—C30	−78.2 (11)
P2—Cu2—Cu3—Br1	64.93 (14)	C19—P2—C25—C30	28.3 (11)
S2—Cu2—Cu3—Br1	162.38 (9)	Cu2—P2—C25—C30	152.3 (9)
S1—Cu2—Cu3—Br1	−98.30 (7)	C30—C25—C26—C27	2.4 (18)
W1—Cu2—Cu3—Br1	−129.67 (4)	P2—C25—C26—C27	−179.1 (10)
Cu1—Cu2—Cu3—Br1	−66.50 (4)	C25—C26—C27—C28	−5 (2)
P2—Cu2—Cu3—Cu1	131.43 (15)	C26—C27—C28—C29	3 (2)
S2—Cu2—Cu3—Cu1	−131.12 (9)	C27—C28—C29—C30	1 (2)
S1—Cu2—Cu3—Cu1	−31.79 (7)	C28—C29—C30—C25	−3 (2)
Br1—Cu2—Cu3—Cu1	66.50 (4)	C26—C25—C30—C29	1.1 (19)
W1—Cu2—Cu3—Cu1	−63.17 (3)	P2—C25—C30—C29	−177.3 (10)
P1—Cu1—Cu3—P3	−10.4 (2)	C25—P2—C31—C32	−1.1 (10)
S3—Cu1—Cu3—P3	−102.46 (16)	C19—P2—C31—C32	−113.0 (9)
S1—Cu1—Cu3—P3	149.61 (13)	Cu2—P2—C31—C32	123.7 (8)
W1—Cu1—Cu3—P3	−174.87 (13)	C25—P2—C31—C36	174.6 (8)
Br1—Cu1—Cu3—P3	55.41 (12)	C19—P2—C31—C36	62.7 (8)
Cu2—Cu1—Cu3—P3	117.51 (13)	Cu2—P2—C31—C36	−60.6 (9)
P1—Cu1—Cu3—S3	92.05 (15)	C36—C31—C32—C33	−0.3 (16)
S1—Cu1—Cu3—S3	−107.93 (12)	P2—C31—C32—C33	175.3 (9)
W1—Cu1—Cu3—S3	−72.41 (10)	C31—C32—C33—C34	−1 (2)
Br1—Cu1—Cu3—S3	157.87 (10)	C32—C33—C34—C35	1 (2)
Cu2—Cu1—Cu3—S3	−140.03 (10)	C33—C34—C35—C36	1 (2)
P1—Cu1—Cu3—S2	−161.99 (14)	C34—C35—C36—C31	−2 (2)
S3—Cu1—Cu3—S2	105.97 (12)	C32—C31—C36—C35	1.7 (17)
S1—Cu1—Cu3—S2	−1.96 (10)	P2—C31—C36—C35	−174.3 (10)
W1—Cu1—Cu3—S2	33.56 (7)	C49—P3—C37—C38	96.3 (10)
Br1—Cu1—Cu3—S2	−96.16 (8)	C43—P3—C37—C38	−15.8 (10)
Cu2—Cu1—Cu3—S2	−34.06 (7)	Cu3—P3—C37—C38	−141.4 (8)
P1—Cu1—Cu3—W1	164.45 (13)	C49—P3—C37—C42	−82.0 (8)
S3—Cu1—Cu3—W1	72.41 (10)	C43—P3—C37—C42	165.9 (8)
S1—Cu1—Cu3—W1	−35.52 (6)	Cu3—P3—C37—C42	40.3 (9)
Br1—Cu1—Cu3—W1	−129.72 (5)	C42—C37—C38—C39	−0.4 (17)
Cu2—Cu1—Cu3—W1	−67.62 (4)	P3—C37—C38—C39	−178.7 (9)
P1—Cu1—Cu3—Br1	−65.83 (13)	C37—C38—C39—C40	−0.4 (18)
S3—Cu1—Cu3—Br1	−157.87 (10)	C38—C39—C40—C41	1.1 (18)
S1—Cu1—Cu3—Br1	94.20 (7)	C39—C40—C41—C42	−1 (2)
W1—Cu1—Cu3—Br1	129.72 (5)	C40—C41—C42—C37	0.4 (18)
Cu2—Cu1—Cu3—Br1	62.10 (4)	C38—C37—C42—C41	0.3 (15)
P1—Cu1—Cu3—Cu2	−127.93 (13)	P3—C37—C42—C41	178.8 (9)
S3—Cu1—Cu3—Cu2	140.03 (10)	C49—P3—C43—C44	159.5 (7)
S1—Cu1—Cu3—Cu2	32.10 (7)	C37—P3—C43—C44	−92.0 (8)
W1—Cu1—Cu3—Cu2	67.62 (4)	Cu3—P3—C43—C44	31.1 (8)
Br1—Cu1—Cu3—Cu2	−62.10 (4)	C49—P3—C43—C48	−21.4 (9)
O1—W1—S1—Cu1	129.6 (3)	C37—P3—C43—C48	87.1 (8)
S2—W1—S1—Cu1	−107.92 (8)	Cu3—P3—C43—C48	−149.8 (7)
S3—W1—S1—Cu1	7.60 (9)	C48—C43—C44—C45	−4.3 (14)
Cu3—W1—S1—Cu1	−50.13 (7)	P3—C43—C44—C45	174.8 (8)
Cu2—W1—S1—Cu1	−92.21 (8)	C43—C44—C45—C46	1.0 (17)
O1—W1—S1—Cu2	−138.2 (3)	C44—C45—C46—C47	1.8 (19)
S2—W1—S1—Cu2	−15.71 (9)	C45—C46—C47—C48	−1 (2)
S3—W1—S1—Cu2	99.81 (8)	C46—C47—C48—C43	−1.9 (17)
Cu3—W1—S1—Cu2	42.08 (7)	C44—C43—C48—C47	4.6 (14)
Cu1—W1—S1—Cu2	92.21 (8)	P3—C43—C48—C47	−174.5 (8)
P1—Cu1—S1—W1	−159.08 (10)	C37—P3—C49—C54	−25.7 (11)
S3—Cu1—S1—W1	−7.34 (9)	C43—P3—C49—C54	83.4 (10)
Br1—Cu1—S1—W1	88.78 (5)	Cu3—P3—C49—C54	−147.0 (9)
Cu2—Cu1—S1—W1	73.52 (6)	C37—P3—C49—C50	151.0 (9)
Cu3—Cu1—S1—W1	35.67 (5)	C43—P3—C49—C50	−99.9 (9)
P1—Cu1—S1—Cu2	127.40 (11)	Cu3—P3—C49—C50	29.7 (10)
S3—Cu1—S1—Cu2	−80.86 (9)	C54—C49—C50—C51	0.3 (17)
W1—Cu1—S1—Cu2	−73.52 (6)	P3—C49—C50—C51	−176.6 (9)
Br1—Cu1—S1—Cu2	15.26 (7)	C49—C50—C51—C52	−0.4 (19)
Cu3—Cu1—S1—Cu2	−37.85 (7)	C50—C51—C52—C53	0.0 (19)
P2—Cu2—S1—W1	157.46 (9)	C51—C52—C53—C54	0 (2)
S2—Cu2—S1—W1	14.78 (9)	C50—C49—C54—C53	0.0 (17)
Br1—Cu2—S1—W1	−90.44 (6)	P3—C49—C54—C53	176.8 (10)
Cu3—Cu2—S1—W1	−32.79 (6)	C52—C53—C54—C49	0 (2)
